# Grip strength but not stair climb power is associated with injurious falls in middle-aged and older women: The Study of Women’s Health Across the Nation (SWAN)

**DOI:** 10.1007/s11657-026-01655-3

**Published:** 2026-03-04

**Authors:** Nina Z. Heilmann, Kristine M. Ruppert, Jimmie E. Roberts, Carrie A. Karvonen-Gutierrez, Kelley Pettee Gabriel, Kelly R. Ylitalo, Bradley C. Nindl, Jane A. Cauley, Elsa S. Strotmeyer

**Affiliations:** 1 Department of Epidemiology, School of Public Health, University of Pittsburgh, Pittsburgh, PA USA; 2 Hinda and Arthur Marcus Institute for Aging Research, Hebrew SeniorLife, Boston, MA USA; 3 Department of Epidemiology, School of Public Health, University of Michigan, Ann Arbor, MI USA; 4 Department of Epidemiology, School of Public Health, University of Alabama at Birmingham, Birmingham, AL USA; 5 Department of Public Health, Robbins College of Health and Human Sciences, Baylor University, Waco, TX USA; 6 Neuromuscular Research Laboratory/Warrior Human Performance Research Center, Department of Sports Medicine and Nutrition, University of Pittsburgh, Pittsburgh, PA USA

**Keywords:** Injurious falls, Grip strength, Stair climb power, Community-dwelling, Women

## Abstract

***Summary*:**

We examined both muscle strength and power in relation to non-injurious and injurious falls in older women. Higher grip strength, but not stair climb power, was associated with lower odds of both fall outcomes. Findings highlight muscle strength as a potential target for fall prevention strategies in older women.

**Background:**

Falls are the leading cause of injury and injury death in older women, with an increase in fall prevalence during midlife. While muscle strength and power may be modifiable risk factors for falls, associations of both muscle strength and power with injurious falls have not been investigated together in a study of community-dwelling middle-aged and older women.

**Methods:**

In the Study of Women’s Health Across the Nation (SWAN), muscle function and falls were measured in 2015–2017 and 2021–2023 (6.6 ± 0.3 years follow-up). Muscle function tests included grip strength (kg/weight(kg)) and stair climb power (W/weight(kg)). Self-reported falls in the past year were categorized as no falls, non-injurious falls, or injurious falls (fractured bone, hit/injured head, sprain/strain, bruises, bleeding, other). Generalized estimating equations were used to model associations between time-varying muscle function measures and fall outcomes adjusted for demographics, body size, lifestyle factors, and multimorbidities.

**Results:**

Among 1710 women (age 65.0 ± 2.7 years), 28% reported injurious falls and 16% reported non-injurious falls during the study period. Average declines were -1.37%/year for stair climb power and -0.90%/year for grip strength. In final models, a 1 standard deviation (0.10 kg/kg) higher grip strength was associated with 18% lower odds of non-injurious falls (OR = 0.82, 95% CI 0.69–0.98) and 19% lower odds of injurious falls (OR = 0.81, 95% CI 0.70–0.94). Stair climb power was not associated with either fall outcome.

**Conclusions:**

Muscle strength may be a potential target for musculoskeletal interventions to reduce fall and fall injury risk in older women.

## Introduction

Falls are the leading cause of unintentional injury and injury death among adults aged 65 years and older [1] and fall-related mortality increased nearly four-fold from 1999 to 2020 [2]. Women are more likely than men to experience fall-related injuries [3] and hospitalizations [4]. In women, fall prevalence rises sharply from 9% in 40–44 year-olds to 30% in 60–64 year-olds, compared to only a 1% increase in men during the same age period [5]. Women also experience worse fall-related outcomes, including higher mortality, than middle-aged men [6], suggesting middle age as a critical period for fall prevention interventions in women.

With accelerated bone loss in middle age due to menopause, women have heightened fracture risk and make up 71% of all fractures among adults aged ≥ 50 years [7, 8]. While fractures are among the most severe fall-related injuries, 60% of medically attended nonfatal injuries in older adults are milder injuries (i.e., bruises, strains, sprains, etc.) [9]. Few studies have included these mild fall injuries as outcomes [10], which may be important due to their significant contribution to healthcare utilization, potential as precursors to more serious injuries in the future, and association with fear of falling-related activity reduction.

Muscle function, which includes muscle strength (force) and power (force*velocity), may be a modifiable risk factor for injurious falls. However, few studies have investigated this association in women; these prior studies focused on fall-related fractures [11, 12]. There is a need for studies of muscle function among midlife women as they transition into older ages that incorporate less severe fall-related injuries, as these may precede the more serious fall-related injuries at older ages. Additionally, existing studies included only a single measure of muscle function, which was typically muscle strength, and no studies included muscle power. Muscle power has been shown to decline earlier and faster than muscle strength [13] and therefore may be an earlier indicator of fall injury risk.

Studies that include multiple measures of muscle strength and power are needed. The stair climb power test is a valid and reliable measure of leg power in older adults that has not been studied as a predictor of fall injury [14]. As a weight-bearing test that incorporates dynamic balance, the stair climb power test may better reflect mobility than seated measures of leg strength and power or upper-extremity strength tests. Grip strength is used in many definitions of sarcopenia and is considered a marker of overall musculoskeletal function in aging populations [15, 16]. Our aim was to evaluate the associations of stair climb power and grip strength with non-injurious and injurious falls collected at two time points over 6.6 years in early older-aged women. We hypothesized that higher stair climb power and grip strength would be associated with lower odds of non-injurious and injurious falls.

## Methods

### Study population

The Study of Women’s Health Across the Nation (SWAN) is an ongoing, multisite, multiethnic, longitudinal cohort study of the menopause transition and healthy aging [17]. Starting in 1996–1997, women were eligible if they were age 42–52 years with intact uterus and at least one ovary, and were not using hormone therapy, were not pregnant, lactating, or breastfeeding, and had ≥ 1 menstrual period in the previous three months. A total of 3302 women enrolled at seven sites across the United States: Boston, MA; Chicago, IL; Pittsburgh, PA; Detroit-area, MI; Oakland, CA; Los Angeles, CA; and Hudson County, NJ. Each site enrolled both White women and non-White racial and ethnic groups including Black (Boston, Pittsburgh, Detroit-area, Chicago), Japanese (Los Angeles), Chinese (Oakland), and Hispanic (Newark) women. Participants provided written informed consent, and all protocols received approval from the institutional review board at each participating site.

**Fig. 1:**
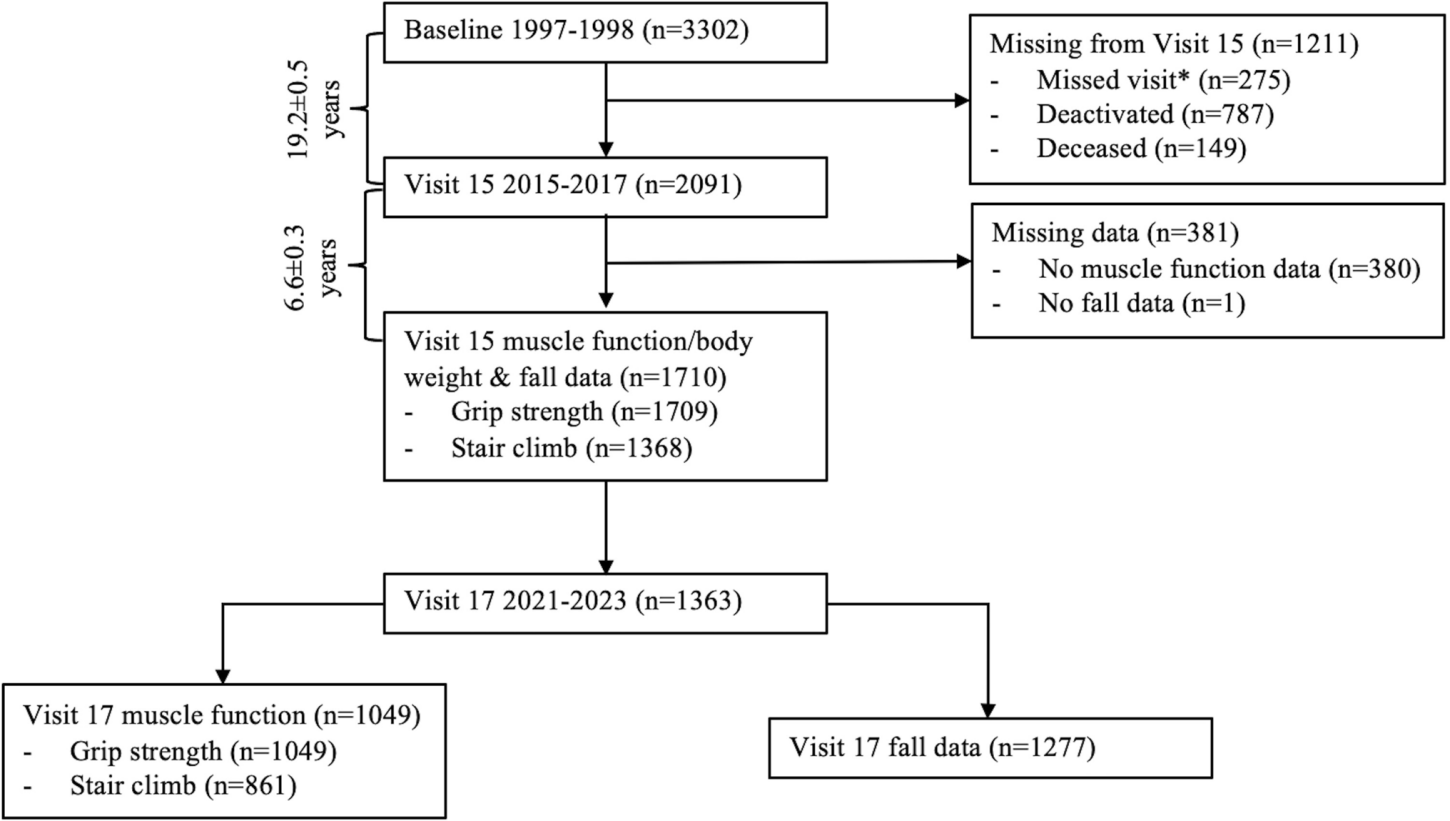
Flowchart of sample selection for participants with muscle function and falls data in the Study of Women’s Health Across the Nation (SWAN). *Reasons for missed visit include respondent refused visit, non-response, unable to schedule visit in collection window, and respondent moved from study area

Of 2091 women who participated in the Visit 15 follow-up exam (2015–2017; age range 60–72 years), 1710 (82%) completed fall assessments and at least one muscle function test (grip strength or stair climb tests). Women self-reported fall history (*n* = 1277) and performed muscle function tests (*n* = 1049) again during the Visit 17 follow-up exam (2021–2023; age range 66–79 years) an average of 6.6 ± 0.3 years later. We included the 1710 women with data from at least one muscle function test and falls assessment at Visit 15 in our analytic sample (Fig. 1).

### Grip strength

Grip strength (kg) was measured at each study visit from 3 trials of each hand using a Baseline^Ⓡ^ hydraulic hand dynamometer. Participants were in a seated position with their elbow bent at a 90° angle. The maximum value of all trials normalized by body weight was used in analyses (kg/kg) to account for the influence of body size on absolute strength and ensure appropriate parameterization of grip strength.

### Stair climb power

The timed stair climb assessment was comprised of four standard stairs (10 inches deep and 6 inches in height). Participants were instructed to ascend and descend the stairs three times at their typical pace without stopping, using the handrail for support if needed [18]. The fastest ascend time (seconds) from 3 laps was used to calculate stair climb power using the formula: power (W) = ((body weight in kg) x (9.8 m/s^2^) x (stair height in m))/(time in seconds) [19]. Stair climb power normalized by body weight was used in analyses (W/kg) to reflect functional power relative to the mechanical demands of moving one’s own body.

The timed stair climb assessment was not completed for participants if they refused, were physically unable, reported knee injury or pain, or the interviewer deemed it unsafe. The Boston, MA and Los Angeles, CA sites did not participate in the timed stair climb assessment.

### Assessment of fall outcomes

Falls were assessed at Visit 15 and Visit 17 by self-report from the question: “In the past year, have you fallen and landed on the floor or ground (or fallen and hit an object like a table or stair)?” If affirmative, two follow-up questions were asked: “How many times have you fallen in the past year?” and “During the past year, were you injured in any of these falls?” If they reported an injury, they were asked to report the type of injury, including broken or fractured bone, hit or injured head, sprain or strain, bruises, bleeding, or other kind of injury. A 3-level fall outcome variable was defined for each study visit, categorized as: 1) no falls, 2) ≥ 1 fall but no fall injuries (i.e., “non-injurious falls”), and 3) ≥ 1 fall with ≥ 1 fall injury (i.e., “injurious falls”). Additionally, a cumulative fall outcome variable was constructed using the same categories by integrating fall data from Visit 15 to Visit 17, assigning each participant to the highest severity level recorded across visits.

### Potential confounders

Covariates from Visit 15 included age (years), self-reported race/ethnicity, cigarette smoking status (never/former/current, combined to never vs. former/current for analyses), alcohol intake frequency (< 1/month, 1/month to 2/week, > 2/week), hormone use since the last study visit (yes/no), leg pain in the last 3 months (yes/no), and physician-diagnosed self-reported conditions (yes/no; high blood pressure, diabetes, arthritis/osteoarthritis, osteoporosis, cancer, and cardiovascular [CVD] conditions: stroke, heart failure, and heart attack/angina). Depressive symptoms were assessed using the Center for Epidemiological Studies Depression Scale (CES-D; score ≥ 16) [20].

Weight (kg) was measured without shoes and in light clothing using digital or balance beam scales. Height (cm) was measured without shoes using a fixed stadiometer. Weight and height were used to calculate body mass index (kg/m^2^). A standard mercury sphygmomanometer was used to measure average blood pressure with participants seated and feet flat on the floor for at least 5 min prior to measurement. Self-reported physical activity in the past year across three domains (sports/exercise, active living, household/caregiving) was assessed using the Kaiser Physical Activity Survey (KPAS), with domain-specific intermediate scores ranging from 1 to 5, with higher values indicating greater physical activity. Domain-specific scores were summed to derive the total physical activity score, ranging from 3–15 [21]. Medication data were collected by asking women to bring in all medications used in the last 30 days. Fall-risk increasing drug (FRID) use included antidepressants, antipsychotics, opioids, anticonvulsants, antihypertensives, muscle relaxants, sedative hypnotics, benzodiazepines, and antihistamines [22, 23]. To evaluate cognitive function (i.e., working memory) [24], the letter number sequencing (LNS) test was used, in which participants were read a combination of numbers and letters and were asked to recall the numbers first in ascending order and then the letters in alphabetical order in three separate trials, with scores ranging from 0 to 21.

## Statistical analysis

Sample characteristics at Visit 15, including muscle function, were compared across levels of the cumulative fall outcome variable using t-tests, Wilcoxon rank-sum tests, or χ^2^ tests, as appropriate. Annualized percent change in muscle function measures was calculated by dividing the percent change from Visit 15 to Visit 17 by follow-up time (in years) between visits:

$$\left[\frac{Visit \,17-Visit\, 15}{Visit\, 15}\times 100\right]\div follow-up\, time$$

Generalized estimating equations (GEEs) with a nominal multinomial distribution and an independent correlation structure were used to model the associations between time-varying muscle function measures and the 3-level fall outcome using repeated assessments of muscle function and falls at Visit 15 and Visit 17. Muscle function measures were entered in separate models as continuous variables. A time interaction term with muscle function measures was tested in all models to determine whether the association between muscle function and odds of non-injurious and injurious falls changed between Visit 15 and Visit 17.

Covariates from Visit 15 were entered sequentially in the order of demographics (age, race/ethnicity), body size (height), lifestyle factors (smoking status, alcohol intake frequency, physical activity), and comorbidities (leg pain, high blood pressure, diabetes, arthritis/osteoarthritis, osteoporosis, cancer, CVD conditions, depressive symptoms, FRID use, hormone use, and LNS test score). Age was forced into models regardless of significance. Other covariates that were significant at ɑ < 0.10 were retained in final models. Since total physical activity differed significantly between fall groups, but the exercise/sports specific domain did not, total physical activity was used in models. Significant covariates from Visit 15 were also tested as time-varying by including their Visit 17 values. Model fit was evaluated using Quasi-likelihood under the Independence model Criterion (QIC/QIC_u_) to assess the inclusion of time-varying covariates. If a covariate was significant in one muscle function model, we forced it into the other model so that final models for each muscle function measure were adjusted for the same set of covariates.

We conducted several sensitivity analyses. Since time-varying GEE models include women with muscle function data available from at least one time point, and some study sites did not participate in stair climb power assessments, we reran models among a subset of women with complete grip strength and stair climb power data at both Visit 15 and Visit 17 (*n* = 843). Then, to account for potential confounding by study site, models were rerun with adjustment for study site instead of race/ethnicity since some racial and ethnic groups were exclusive to single sites. We also evaluated the associations between low grip strength (< 20 kg) and fall outcomes, as this threshold is commonly used in sarcopenia definitions and has been associated with falls [25]. Analyses were performed on the dataset provided in February 2025, using SAS (version 9.4) and plots were created in R (version 4.4.1).

## Results

### Unadjusted results

At Visit 15, 316 (18.5%) women reported injurious falls, 195 (11.4%) reported non-injurious falls, and 1199 (70.1%) reported no falls in the past year. Of 1277 women with falls data at Visit 17, 241 (18.9%) reported injurious falls, 193 (15.1%) reported non-injurious falls, and 843 (66.0%) reported no falls in the past year. Most women with injurious falls were non-recurrent fallers (reporting one fall in the past year) at both Visit 15 (59.2%) and Visit 17 (55.6%). Cumulatively from Visit 15 to Visit 17, 483/1710 (28.2%) women reported injurious falls, 269 (15.7%) reported non-injurious falls, and 958 (56.0%) reported no falls.

Comparisons of sample characteristics across categories of the cumulative fall outcome variable found that compared to women with no falls, women with non-injurious and injurious falls had statistically significant differences in the overall distribution of racial and ethnic groups, were more likely to have history of arthritis/osteoarthritis, osteoporosis, CVD conditions, and depressive symptoms, and had lower weight-corrected grip strength (Table 1; p < 0.05). Compared to women with no falls, women with injurious falls also were more likely to have leg pain and higher LNS test scores (Table 1; p < 0.05). Compared to women with no falls, women with non-injurious falls also had higher body weight, were more likely to have ever smoked cigarettes, have history of cancer, and had lower absolute grip strength (Table 1; p < 0.05). There were no significant differences in absolute grip strength among those with injurious falls vs. no falls. Absolute and weight-corrected stair climb power did not differ significantly among women with non-injurious or injurious falls compared to those with no falls. Compared to those with non-injurious falls, women with injurious falls had higher self-reported physical activity, were less likely to have history of cancer, and had higher LNS test scores (Table 1; p < 0.05). No significant differences in absolute or weight-corrected grip strength or stair climb power were observed between women with non-injurious and injurious falls.

**Table 1:** Descriptive characteristics of participants by cumulative fall outcome status in the Study of Women’s Health Across the Nation (SWAN)

	Overall (*N* = 1710)	No falls (*n* = 958)	Non-injurious falls (*n* = 269)	Injurious falls (*n* = 483)
Age, years	65.0 ± 2.7	65.0 ± 2.7	65.1 ± 2.7	65.0 ± 2.7
Age ≥ 65 years	937 (55)	527 (55)	156 (58)	254 (53)
Race/ethnicity^a,b^				
Black	460 (27)	282 (29)	78 (29)	100 (21)
White	815 (48)	422 (44)	114 (42)	279 (58)
Chinese	173 (10)	107 (11)	20 (7)	46 (10)
Hispanic	94 (6)	50 (5)	28 (10)	16 (3)
Japanese	168 (10)	97 (10)	29 (11)	42 (9)
Weight, kg	75.6 ± 19.4	74.8 ± 19.7	77.6 ± 19.6^a^	76.0 ± 18.5
Height, cm	160.7 ± 6.6	160.5 ± 6.6	160.9 ± 6.8	160.9 ± 6.3
Body mass index, kg/m^2^	29.2 ± 7.1	28.9 ± 7.2	29.9 ± 7.1	29.2 ± 6.8
Ever smoked, yes	665 (39)	347 (36)	120 (45)^a^	198 (41)
Alcohol intake				
< 1/month	855 (50)	481 (50)	150 (56)	224 (46)
1/month—2 +/week	407 (24)	240 (25)	56 (21)	111 (23)
2 +/week	426 (25)	224 (23)	61 (23)	141 (29)
Physical activity, 3–15	7.5 ± 1.8	7.5 ± 1.8	7.3 ± 1.9	7.7 ± 1.8^b^
Sports/exercise domain, 1–5	2.8 ± 1.0	2.8 ± 1.0	2.8 ± 1.1	2.9 ± 1.0
FRID use, yes	1083 (63)	591 (62)	175 (65)	317 (66)
Hormone use since last visit, yes	97 (6)	52 (5)	13 (5)	32 (7)
Leg pain last 3 months, yes	500 (29)	249 (26)	83 (31)	168 (35)^a^
High blood pressure hx, yes	938 (55)	534 (56)	151 (56)	253 (52)
Diabetes hx, yes	355 (21)	189 (20)	67 (25)	99 (21)
Arthritis/osteoarthritis hx, yes	1077 (63)	560 (58)	185 (69)^a^	332 (69)^a^
Osteoporosis hx, yes	410 (24)	207 (22)	76 (28)^a^	127 (26)^a^
Cancer hx, yes	208 (12)	106 (11)	46 (17)^a^	56 (12)^b^
CVD conditions hx, yes	146 (8)	61 (6)	37 (14)^a^	48 (10)^a^
Depressive symptoms, yes	212 (12)	88 (9)	42 (16)^a^	82 (17)^a^
Systolic blood pressure, mmHg	123.3 ± 15.3	123.5 ± 15.6	124.0 ± 16.1	122.7 ± 14.3
Diastolic blood pressure, mmHg	73.7 ± 9.2	73.8 ± 9.1	73.7 ± 9.2	73.6 ± 9.6
LNS test score, 0–21	9.5 ± 2.6	9.3 ± 2.6	9.3 ± 2.6	10.1 ± 2.5^a,b^
Muscle function measures				
Grip strength (kg/kg body weight)	0.34 ± 0.10	0.35 ± 0.10	0.33 ± 0.10^a^	0.34 ± 0.09^a^
Stair climb power (W/kg body weight)	2.17 ± 0.52	2.18 ± 0.51	2.12 ± 0.56	2.19 ± 0.53
Grip strength (kg)	24 [20, 28]	24 [22, 28]	24 [20, 28]^a^	24 [20, 28]
Stair climb power (W)	154.6 [122.8, 186.4]	152.9 [121.8, 182.5]	153.7 [124.3, 187.5]	158.9 [123.9, 192.1]

Values presented are mean ± SD or median [IQR] for continuous variables and n (%) for categorical variables

*CVD* cardiovascular, *FRID* fall risk increasing drug, *hx* history, *LNS* letter number sequencing

*P*-value < 0.05 for pairwise comparisons using t-tests or Wilcoxon rank-sum tests for continuous variables and $\chi$ ^2^ tests for categorical variables with ^a^no falls as referent group or ^b^non-injurious falls as referent group

**Fig. 2:**
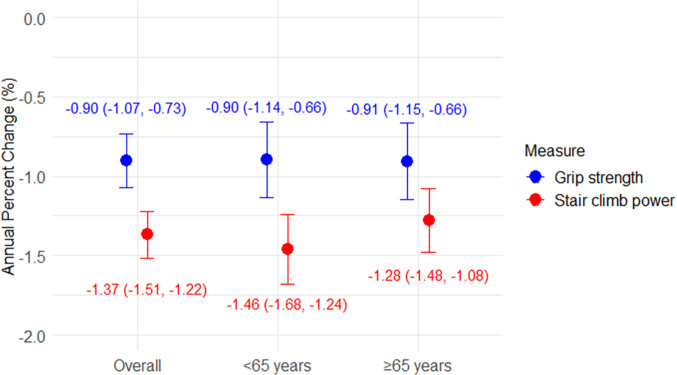
Average annual percent changes (with 95% confidence intervals) in muscle function measures from Visit 15 to Visit 17 over 6.6 ± 0.3 years of follow-up

During follow-up (6.6 ± 0.3 years), both muscle function measures declined on average. Mean (95% CI) changes were −1.37 (−1.51 to −1.22) % per year for stair climb power and −0.90 (−1.07 to −0.73) % per year for grip strength (Fig. 2).

### Adjusted results

In fully adjusted models, a 1 SD (0.10 kg/kg) higher grip strength was associated with 18% lower odds of non-injurious falls (odds ratio [OR] 0.82, 95% CI 0.69–0.98) and 19% lower odds of injurious falls (OR 0.81, 95% CI 0.70–0.94) from Visit 15 to Visit 17 compared to no falls (Table 2). A 1 SD (0.50 W/kg) higher stair climb power was associated with lower odds of non-injurious (OR 0.89, 95% CI 0.70–1.13) and injurious falls (OR 0.82, 95% CI 0.67–1.01) from Visit 15 to Visit 17, though results did not reach statistical significance (Table 2). There were no significant associations between grip strength or stair climb power and the odds of injurious falls vs. non-injurious falls (data not shown).

**Table 2:** Associations of muscle function with fall outcomes from Visit 15 to Visit 17 in the Study of Women’s Health Across the Nation (SWAN)

	*n*	Odds Ratio (95% CI)		
		No falls	Non-injurious falls	Injurious falls
*Grip strength (per SD)*	1710	*Ref*	0.82 (0.69, 0.98)^a^	0.81 (0.70, 0.94)^a^
*Stair climb power (per SD)*	1384	*Ref*	0.89 (0.70, 1.13)	0.82 (0.67, 1.01)

^*a*^*P* < 0.05; models adjusted for age (years), race and ethnicity, height (cm), ever smoker (yes/no), physical activity (3–15), arthritis/osteoarthritis (yes/no), osteoporosis (yes/no), cancer (yes/no), CVD conditions (yes/no), depressive symptoms (yes/no), letter number sequencing test score (0–21)

1 SD grip strength = 0.10 kg/kg body weight

1 SD stair climb power = 0.50 W/kg body weight

### Sensitivity analyses

Among a subset of women with complete muscle function data at both Visit 15 and Visit 17 (*n* = 843; Table 3), higher grip strength remained significantly associated with lower odds of injurious falls compared to no falls, with a stronger effect size observed. The association between grip strength and non-injurious falls vs. no falls remained similar in magnitude but was no longer statistically significant. Associations between stair climb power and both non-injurious and injurious falls remained non-significant. Results were consistent when adjusting for study site instead of race/ethnicity (data not shown). Low grip strength (< 20 kg) was not significantly associated with non-injurious or injurious falls (data not shown).

**Table 3:** Associations of muscle function with fall outcomes from Visit 15 to Visit 17 among those with complete muscle function data in the Study of Women’s Health Across the Nation (SWAN)

	*n*	Odds Ratio (95% CI)		
		No falls	Non-injurious falls	Injurious falls
*Grip strength (per SD)*	843	*Ref*	0.83 (0.65, 1.06)	0.72 (0.58, 0.91)^a^
*Stair climb power (per SD)*	843	*Ref*	0.86 (0.66, 1.14)	0.82 (0.65, 1.05)

^*a*^*P* < 0.05; models adjusted for age (years), race and ethnicity, height (cm), ever smoker (yes/no), physical activity (3–15), arthritis/osteoarthritis (yes/no), osteoporosis (yes/no), cancer (yes/no), CVD conditions (yes/no), depressive symptoms (yes/no), letter number sequencing test score (0–21)

1 SD grip strength = 0.10 kg/kg body weight

1 SD stair climb power = 0.50 W/kg body weight

## Discussion

We found that higher grip strength, but not stair climb power, was significantly associated with lower odds of injurious falls over 6.6 ± 0.3 years of follow-up. This finding aligns with the well-established role of grip strength as a key marker of musculoskeletal health in older adults, as it is widely used in definitions of sarcopenia and has been linked to falls, frailty, disability, and mortality [15, 16, 25].

Our observed association between grip strength and injurious falls may be explained by several factors. Muscle strength is positively correlated with muscle mass [26], which may help protect against injury by reducing impact severity during a fall [27]. Stronger muscles also exert greater mechanical stress on bones, which can improve bone strength and lower fracture risk [28]. Grip strength is also commonly used as a surrogate marker of overall musculoskeletal health and may reflect systemic functional declines that could contribute to fall risk, frailty, and injury susceptibility [16]. Biomechanical studies of falls indicate a role of upper-extremity muscle strength in injury prevention, such as hitting or grabbing onto an object to avoid direct impact on fragile parts of the body [27]. Since falls occur when the center of mass moves too far outside the base of support, effective change-in-support balance recovery often requires the ability to quickly reach and grasp a support when corrective stepping is insufficient [29]. Further research is needed to understand the interplay between muscle strength, muscle mass, and fall-related injury mechanisms to better understand why grip strength, but not lower-extremity power, was associated with injurious falls in this cohort.

Few existing studies have evaluated muscle function associations with injurious falls, and most included only one muscle function measure and focused on serious injuries. One cross-sectional study of 356 older adults in Australia (age ≥ 65 years, 75% women) found that low grip strength (< 35.5 kg for men, < 20 kg for women) was significantly associated with fall-related fractures (OR 2.26, 95% CI 1.17–4.36), adjusted for age, sex, BMI, comorbidities, and other covariates [30]. Another cross-sectional study of 2323 older adults in South Korea (age 70–84 years, 52% women) found that low grip strength (< 18 kg in women) was not significantly associated with fall-related fractures in women, adjusted for age, BMI, comorbidities, and other covariates [11]. We observed non-significant associations between low grip strength (< 20 kg) and injurious falls, though we found significant associations when grip strength was analyzed as a continuous variable. These inconsistent findings across studies may be related to different quantification of grip strength (i.e., continuous vs. various cut points), the inclusion of men in the first study, and other population differences.

For lower-extremity muscle function, a study of 273 middle-aged women in Tasmania found that neither baseline nor 5-year change in leg strength (from a dynamometer) was significantly associated with self-reported injurious falls (no definition provided), adjusted for age, weight, height, and lifestyle factors [12]. These null findings for lower-extremity muscle strength are consistent with our lack of associations for lower-extremity muscle power. Existing studies are limited by inconsistent definitions of injurious falls (i.e., fractures vs. minor injuries), which may yield different results depending on the severity of injuries included. Low event occurrence (< 10%) [11, 12, 31] in the few existing studies of fall-related fractures may impact statistical power and highlights the importance of including more common milder injuries in injurious fall definitions.

To our knowledge, our study is the first to examine upper- and lower-extremity measures of muscle strength and power in relation to injurious falls in middle-aged and older women. One prior study among 5178 older men (age 73.4 ± 5.7 years) evaluated the associations of grip strength and leg extension power (from the Nottingham Power Rig) with injurious falls (fracture, head injury, sprain/strain, bruise/bleeding, other) self-reported prospectively over 9 years [32]. Authors found that in men, lower grip strength/body weight (per SD) and leg power/body weight (per SD) were independently associated with higher odds of injurious falls (OR 1.11, 95% CI 1.05–1.17; OR 1.13, 95% CI 1.06–1.20, respectively), adjusted for age, race, height, and comorbidities. These findings suggest that both upper-extremity strength and lower-extremity power may be important for the prediction of injurious falls in men. We found that grip strength and stair climb power showed similar inverse associations with injurious falls (ORs 0.81–0.82), though only the grip strength association was statistically significant. However, sex differences in muscle strength, power, and fall/fall injury rates, as well as hormonal changes during the menopause transition that exacerbate muscle and bone loss in women [33] limit the comparability of these studies.

Our finding that only grip strength, and not stair climb power, was significantly associated with injurious falls was unexpected, as muscle power may decline earlier and more rapidly than muscle strength in older adults [34]. In our cohort, stair climb power showed greater annual declines than grip strength, supporting this premise. The more pronounced decline in muscle power is likely attributed to its neuromuscular properties (e.g., reduced motor unit firing frequency, preferential wasting of type II/fast-twitch fibers) [35, 36], which are critical for rapid postural adjustments after a perturbation to balance [27]. Since the sample size was smaller for the stair climb than the grip strength test, we may have had lower statistical power to detect an association. It is also important to note that we used a *typical pace* stair climb power test rather than instructing women to perform the test as fast as possible. This approach may not have elicited maximal effort and could have limited our ability to detect associations between muscle power and injurious falls. A maximal-effort stair climb power test or alternative assessments of muscle power may better discriminate fall risk in generally healthy middle-aged and older women. Additionally, stair climb power reflects *functional* power, which incorporates physical performance (i.e., balance and coordination), rather than measuring pure muscle power. Other maximal tests, such as the Keiser pneumatic leg press or Nottingham power rig, that isolate muscle power (i.e., power is generated by one action) may yield different results. These mechanisms and considerations suggest that muscle power may play a key role in fall prevention, though its relationship with injurious falls warrants further investigation using targeted power assessments.

This study has several strengths. The longitudinal design provided insight into how muscle function relates to fall risk in women over 6.6 ± 0.3 years of follow-up. To our knowledge, this is the first to evaluate multiple measures of upper-extremity muscle strength and lower-extremity muscle power in relation to both non-injurious and injurious falls in women > 60 years, with many of the cohort younger than typical cohorts of older women. Additionally, our study included a well-characterized, racially and ethnically diverse cohort with comprehensive assessments of muscle function, comorbidities, lifestyle factors, cognitive function, and other relevant covariates. Another strength is our inclusive definition of injurious falls, which incorporated both minor and serious injuries. Given that 30–50% of falls result in minor injuries and 10% lead to serious injuries [37], and that milder injuries account for 60% of medically attended nonfatal injuries [9], this broader classification may better capture the full impact of fall-related injuries. While most women with injurious falls were non-recurrent fallers (~ 60%), there is still a large proportion of women who were recurrent fallers without injury, and future research should investigate whether one-time injurious fallers differ from recurrent injurious fallers.

There are also several limitations of this study. First, missing data may not have been completely at random, as assumed in GEE models. Stair climb test exclusions (refusal, physical inability, knee injury or pain, or safety concerns) may have removed those at highest risk of injurious falls, potentially attenuating the observed associations. However, only 4.4% of women were missing stair climb data, and our results were largely unchanged in sensitivity analyses restricted to women with complete muscle function data, suggesting minimal bias from missingness. Second, we were unable to evaluate associations by specific injury type due to the low prevalence of certain injuries (e.g., only 2% of women experienced fall-related fractures). Lastly, generalizability may be limited as our sample consisted of women who were able to attend in-person visits and complete muscle function assessments. Future studies should replicate these findings in other populations, including men, to better understand potential sex differences in the relationship between muscle function and injurious fall risk.

## Conclusion

Grip strength may be a stronger predictor of injurious fall risk than stair climb power, potentially reflecting broader aspects of musculoskeletal health. Therefore, strength may serve as a target for interventions aimed at reducing injurious falls in older women.

## Data Availability

SWAN provides access to public use datasets that include data from SWAN screening, the baseline visit and follow-up visits (https://agingresearchbiobank.nia.nih.gov/). To preserve participant confidentiality, some, but not all, of the data used for this manuscript are contained in the public use datasets. A link to the public use datasets is also located on the SWAN web site: http://www.swanstudy.org/swan-research/data-access/. Investigators who require assistance accessing the public use dataset may contact the SWAN Coordinating Center at the following email address: swanaccess@edc.pitt.edu.
